# Strength, Elastic Properties and Fiber–Matrix Interaction Mechanism in Geopolymer Composites

**DOI:** 10.3390/polym14061248

**Published:** 2022-03-20

**Authors:** Susana P. Arredondo, Ramón Corral, A. Valenciano, Carlos A. Rosas, Jose M. Gómez, Teresita J. Medina, Magnolia Soto, Jesús M. Bernal

**Affiliations:** 1 Faculty of Engineering, Autonomous University of Sinaloa, Los Mochis 81223, Mexico; paola.arredondo@uas.edu.mx (S.P.A.); aholibama.valenciano.fim@uas.edu.mx (A.V.); carlos.arc@uas.edu.mx (C.A.R.); teresita.medina@uas.edu.mx (T.J.M.); msotofelix@uas.edu.mx (M.S.); 2 Barcelona School of Building Construction, Polytechnic University of Catalonia, 08028 Barcelona, Spain; josemanuel.gomez@upc.edu; 3 School of Engineering, Autonomous University of Sinaloa, Mazatlán 82017, Mexico; jmbernalc@uas.edu.mx

**Keywords:** geopolymer, fibers, resilience, elastic modulus, stress–strain, SEM

## Abstract

The current geopolymers have limited mechanical strength against the effect of tension, which makes them susceptible to brittle failure. However, owing to their potential as a sustainable construction material, there is growing interest in improving the poor mechanical properties of geopolymers. This study experimentally investigated crucial properties of polypropylene-fiber-reinforced fly ash-based geopolymer composites. The effects of polypropylene fibers (PPF) addition (0.5%, 1.0% and 1.5% by volume) on the mechanical properties of the geopolymer composites were investigated with respect to compressive and flexural strength, deformation behavior of Young’s and shear moduli, and resilience capacity. In addition, scanning electron microscopy was performed to establish the morphology of the geopolymeric matrix and the fiber–matrix interfacial interaction. The addition of PPF significantly increased the flexural strength: compared with the control, at 7 days it was 27% greater for the 0.5% PPF composite and 65% greater for the 1.0% PPF composite. By 14 days it was 31% and 61% greater, respectively. By contrast, the 1.5% PPF composite had lower strength parameters compared with the control because the fiber dispersion increased the porosity. Similar trends were seen for resilience. The SEM observations showed the dispersion of the fibers and helped elucidate the fiber–matrix interaction mechanism.

## 1. Introduction

The need for environmentally friendly construction materials for sustainable development is a pressing environmental issue in the construction industry. The concrete industry is said to be a substantial contributor to global warming owing to the use of Portland cement as the main component in the production of concrete and other cement-based construction materials. The cement industry is responsible for approximately 5–7% of the CO_2_ emissions worldwide [1,2,3]. However, the use of concrete and cement-based composites, as the most widely used construction materials, remains unavoidable for the foreseeable future. In this respect, efforts to find alternatives to Portland cement are necessary. In recent years, there have been considerable developments in a new type of inorganic cementitious material called ‘‘geopolymer’’. Geopolymers are amorphous three-dimensional alumina-silicate binder materials first named and developed by Davidovits in the late 1970s [4]. These materials have been limited to small-scale niche applications due to their low flexural and compressive strength, and they are prone to sudden brittle failure [5,6,7]. However, adding fibers can improve the strength of the resulting product. Studies using different types and amounts of fibers to examine their effect on the behavior of the binder have been conducted using common mechanical tests [8,9,10,11,12,13,14,15].

There are many studies evaluating the mechanical performance of geopolymer materials reinforced with polypropylene fibers (PPF) [16,17]. Ranjbar et al. [18] carried out a comprehensive study to evaluate the short- and long-term impacts of different volume percentages of PPF reinforcement on fly ash-based geopolymer composites. The results showed that the incorporation of up to 3 wt% PPF in the geopolymer paste reduced the shrinkage and enhanced the energy absorption of the composites, depending on the fiber content. It could also reduce the ultimate flexural and compressive strength of the material depending on fiber content. Borges et al. [19] studied the flexural behavior of fly ash-based geopolymer composites reinforced with steel fibers (STF) and PPF. The results showed that heat curing improved the strength properties of the macro-fiber-reinforced geopolymer composite. The PPF did not lead to a significant improvement in the compressive strength, but they improved the indirect tensile and flexural strength to some extent. Xu et al. [20] investigated the mix design and mechanical properties of polyvinyl alcohol fiber (PVAF) reinforced fly ash-based geopolymer composites using two different lengths of the fibers. The results showed that the compressive, flexural and tensile strengths increased compared with the control samples. The authors also concluded that the geopolymers with longer fibers exhibited improved toughness and strength characteristics. Al-mashhadani et al. [21] carried out a mechanical and microstructural characterization of fiber-reinforced fly ash-based geopolymer composites using STF, PPF and PVAF. The results showed a significant increase in the flexural strength. For instance, the 28-day flexural strength of PPF, STF and PVAF composites was 14.6%, 31.45%, and 39.84% higher than the control non-fibrous geopolymer sample, respectively. Fiber addition also slightly improved the compressive strength performance. The 28-day compressive strength composites of the STF and PVAF composites increased by 3.37% and 4.26%, respectively, compared with control. All of the fiber-reinforced composites exhibited a significant improvement in drying shrinkage compared with the control sample. Bhutta et al. [22] studied the characteristics of microfiber-reinforced geopolymer mortars for repair using STF, PPF, PVAF, polyethylene terephthalate fibers and carbon fibers. The results revealed that high-strength, high-modulus steel fibers provided the best response compared with the other fibers irrespective of fiber content and curing regime. The PPF performed better than the PVAF, showing large midpoint deflections that were greater than 150 (i.e., large crack widths). Ranjbar and Zhang [23] reviewed the state-of-the-art of fiber-reinforced geopolymer composites and observed that different properties of the resulting composites can be obtained by varying the material and geometrical properties of fibers, the chemical composition of binder, the casting procedure, and the environmental conditions. The most used synthetic fibers in cementitious matrices are based on PP. The main advantages of this fiber are its low cost, inert characteristics at the high pH of the cementitious environment, ability to limit plastic shrinkage cracking of the matrix, and easy dispersion. Based on the literature review, de Azevedo et al. [24] compared the physical and mechanical performance of geopolymer matrices reinforced with natural fibers (cotton, jute, sisal) with those using synthetic fibers (polypropylene, steel, carbon). The natural fibers were found to be sensitive to the alkaline environment and substances on their surfaces that are not favorable for good adhesion with the matrix, thus, fiber pre-treatment is necessary to overcome this and optimize composite performance. Regarding mechanical performance, composites reinforced with natural fibers showed slightly lower resistance than those made with synthetic fibers.

The performance of fibers in geopolymer matrices is highly dependent on the inherent properties of the fibers, the fiber content, geopolymer precursors, curing conditions and curing time of composite [25,26]. However, the fiber–matrix interface plays the primary role in the overall mechanical properties of composite structures. A strong contact interface has the potential to transfer the load from the matrix to fibers with high load-bearing capacity, while fibers with inert surfaces result in a weak interfacial contact, resulting in interface debonding-based composite failure [27]. However, there is little information on the influence of different fibers on the elastic behavior and fiber–matrix interaction mechanism of geopolymer repair materials. This study investigated the effects of PPF on the elastic behavior of geopolymer repair mortars. The obtained performance characteristics of the PPF-reinforced geopolymer mortars improves our understanding of geopolymers as an alternative repair material.

## 2. Materials and Methods

To prepare the geopolymers, low calcium class F fly ash (FA) with a median particle size of 45 µm was used. The results of the X-ray fluorescence (XRF) analysis (Spectrometer Bruker S4 Pioneer, Billerica, MA, USA) of the FA are shown in Table 1. FA from the José López Portillo coal-fired power plant, located in Coahuila, México, was used because this facility continuously generates this reusable industrial waste.

The fine aggregate was natural silica sand conforming to ASTM C778 [28] in saturated surface dry condition. This aggregate was chosen to avoid variability in the results due to the different types and forms of sand around the world. The alkaline activator solution (AS) was a solution comprising sodium silicate (SS) and sodium hydroxide (NaOH) supplied by Sigma Aldrich, St. Louis, MI, USA. The NaOH was in pellet form at 99% purity. The SS was used in liquid form with a modulus ratio (SiO_2_/Na_2_O) of 2.5 (SiO_2_ = 26.5%; Na_2_O = 10.6% and H_2_O = 62.9%). The PPF was supplied by Cofisa, Monterrey, México and was chosen for its low cost compared to other fibers and because PPF is extensively produced from raw materials or recycled from plastic waste. The physical and mechanical properties of the PPF are shown in Table 2.

### 2.1. Sample Preparation

**Figure 1:**
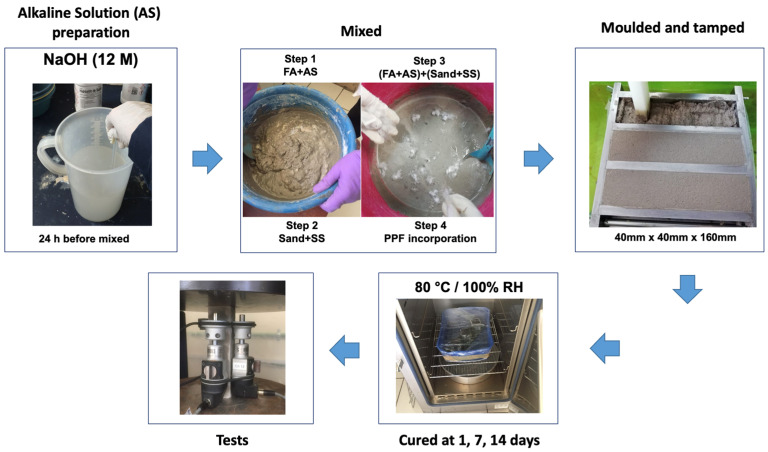
Test sample preparation procedure.

The composition of the geopolymer composite was preliminarily optimized, so that the composite had good strength and adequate workability for fiber dispersion. The final trial composites were prepared with an AS/FA ratio of 0.48 by mass and a sand/FA ratio of 2.75. Four hundred grams of FA was used to prepare each lot of three beams (40 mm × 40 mm × 160 mm). The procedure used to prepare the test samples is shown in Figure 1.

PPF at different volume ratios (0.5%, 1.0% and 1.5%) was slowly added and then mixed with an electrical mixer at low speed until the fibers were thoroughly distributed; this process took approximately 10 min. The mixtures were cast into molds and tamped according to the procedure specified in ASTM C348 [29], sections 9.3.3 and 9.3.4. The samples were identified as follows: G0 (control, without PPF), G1 (0.5% PPF), G2 (1.0% PPF) and G3 (1.5% PPF).

### 2.2. Strength Properties Tests

The flexural behavior was studied using a three-point loading test according to ASTM C348 at a loading rate of 50 N/s. At each curing time, three samples from each mixture were tested, and the results are expressed as the average.

A compressive strength test was performed in accordance with ASTM C349 [30]. Portions of prisms broken in the flexure test were used as the test samples; four samples from each mixture were tested, and the results are expressed as the average. The loading rate was 1400 N/s, in accordance with ASTM C109 [31].

### 2.3. Elastic Properties Tests

To evaluate the elastic properties, prior to the stress–strain test, fundamental resonant frequencies were determined according to ASTM C215 [32] using the prismatic samples in order to determine dynamic Young’s modulus (Ed), dynamic shear modulus (Gd) and Poisson’s ratio (μ).

After the non-destructive testing, three samples (40 mm × 40 mm × 160 mm) were measured with strain gage to record the axial strains produced by compressive stress. The test was conducted in accordance with European standard EN 12390-13 [33] and consisted of three pre-loading cycles from 0.5 MPa to 10% of compressive strength, followed by three loading cycles from 10% to 33% of compressive strength. Static Young’s modulus (Es) was determined from the slope of the stress–strain curve in the linear elastic range (10% to 33% of compressive strength). Resilience (Ur), defined as the ability of an elastic material to absorb energy produced by an external load and release that energy as it springs back to its original shape, was determined as the area under the stress–strain curve from zero to yield stress (33% of compressive strength).

The strength and elastic properties were determined in Automax multitest machine (Controls, Milan, Italy).

### 2.4. Scanning Electron Microscopy (SEM) Observations

**Figure 2:**
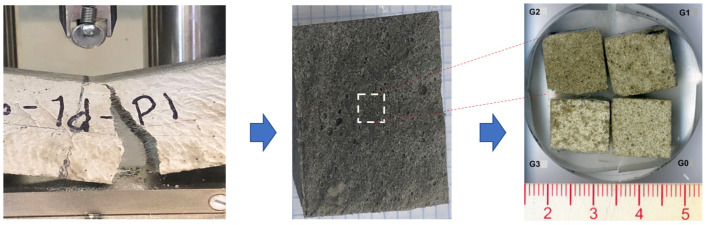
SEM samples embedded in resin and polished.

To carry out the SEM observations, samples (10 mm × 10 mm × 10 mm) were cut from beams used in the flexural test and further prepared (Figure 2). To observe fiber dispersion, matrix morphology and its adherence with the fibers, samples were coated with gold to obtain the necessary conductivity to acquire the backscattered electron images in JEOL JSM-6510 microscope (Jeol Ltd., Tokyo, Japan). To observe fiber dispersion, porosity, microcracks and the interfacial zone between the matrix and fibers and between the matrix and sand, samples were embedded in resin, polished and coated with gold in order to obtain the necessary conductivity to acquire the backscattered and secondary electron images.

## 3. Results and Discussion

### 3.1. Strength Properties

The literature contains conflicting results regarding the compressive strength of fiber-reinforced geopolymer materials. However, it is clear that it depends on different parameters, such as type of mix design, mixing method, fiber type, fiber content, curing conditions and, possibly, the size and type of sample.

**Figure 3:**
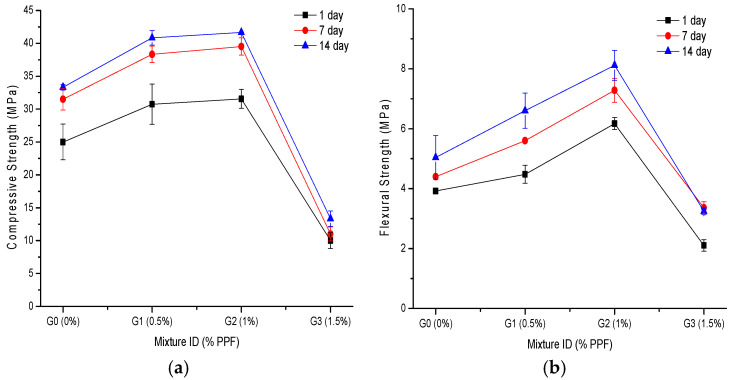
Effect of PPF content at different curing times on (**a**) compressive strength; and (**b**) flexural strength.

The compressive and flexural strength results are shown in Figure 3. Figure 3a shows a significant increase in the compressive strength with increasing fiber content up to 1% (G2 composite). For example, at 14 days the G1 and G2 samples showed 23% and 25% greater compressive strength, respectively, compared with the control sample (G0). Regarding the curing time, 7 days of curing significantly increased the strength compared to 1 day of curing. However, for 14 days, the increase was minimal compared to 7 days. At 1, 7 and 14 days, the G3 samples showed poor results compared with the control sample, showing strength decreases of −60%, −65% and −60%, respectively. A previous study [18], reported that for 7 days of curing, the compressive strength increased when 0.5% PPF was used; however, for higher PPF contents there was a decrease in strength and for curing times greater than 7 days, there was no significant increase in compressive strength. However, another study [17] found that geopolymers containing 0.5 wt% PPF had higher strengths (67.8% and 19.5% for 1 and 3 days, respectively) than control samples. In another study [21], the compressive strength was slightly reduced as the fiber content increased (fiber contents of 0.4%, 0.8% and 1.2%).

In the current study, pieces of beams that were used in the bending test were subsequently used in the compression test, which means that the preferential orientation of the fibers was in the direction of the longitudinal axis of the beams. This is because when the samples are molded, the fibers tend to orient to where the mix flows during compaction or vibration [21]. This means that the application of the axial compression load was perpendicular to the axis of the fibers, which contributed to absorbing part of the fracture energy and mitigating the propagation of cracks that were generated parallel to the direction of the axial compression load [23]. Once a crack contacts the face of a fiber, more energy is needed to fracture the fiber and continue propagation. Hence, the ultimate strength of the composites is increased [25]. This explains the significant increases in compressive and flexural strength that were obtained for composites G1 and G2 when compared with the control sample (G0).

Figure 3b shows that fiber addition achieved a significantly increased flexural strength. For instance, the 7 day G1 and G2 samples had 27% and 65% greater flexural strength compared with the control, and the 14 day G1 and G2 samples were 31% and 61% greater, respectively. At 1, 7 and 14 days, the G3 samples showed poor flexural strength results, decreasing by 46%, 24% and 36%, respectively, compared with the control. Other investigations [17,18,21] have also found that the flexural strength increases with increasing PPF content, up to a certain optimal percentage, and then, an adverse effect occurs. Those studies also showed that the flexural strength did not significantly increase beyond 7 days of curing.

The strength properties depended not only on the NaOH concentration but also on the curing time due to the continuous geopolymerization reaction over a long time. The increased compressive and flexural strength was due to the reaction between Si^4+^ and Al^3+^ ions released from aluminosilicate particles of FA; moreover, this increase occurred because the geopolymeric gel filled in the pores in the geopolymer samples [18,34]. The drastic reduction in compressive and flexural strength in the G3 (1.5% PPF) geopolymer composite can be attributed to poor fiber dispersion, which was observed as difficulties in casting and tamping the material in its fresh state. It is possible that fibers tended to clump together during mixing, entrapping water-filled pores that subsequently turn into voids [22].

### 3.2. Elastic Properties

The various elastic properties are shown in Table 3. The addition of up to 1% PPF improved the elastic behavior compared with the control sample (G0). For example, at 7 days, the Es and ε_y_ of G1 (0.5% PPF) were 25% and 65% greater than those of G0, and the Es and ε_y_ of G2 (1% PPF) were 14% and 72% greater than those of G0, respectively. As in the strength results, the G3 sample showed a drastic reduction in elastic properties with the control.

**Figure 4:**
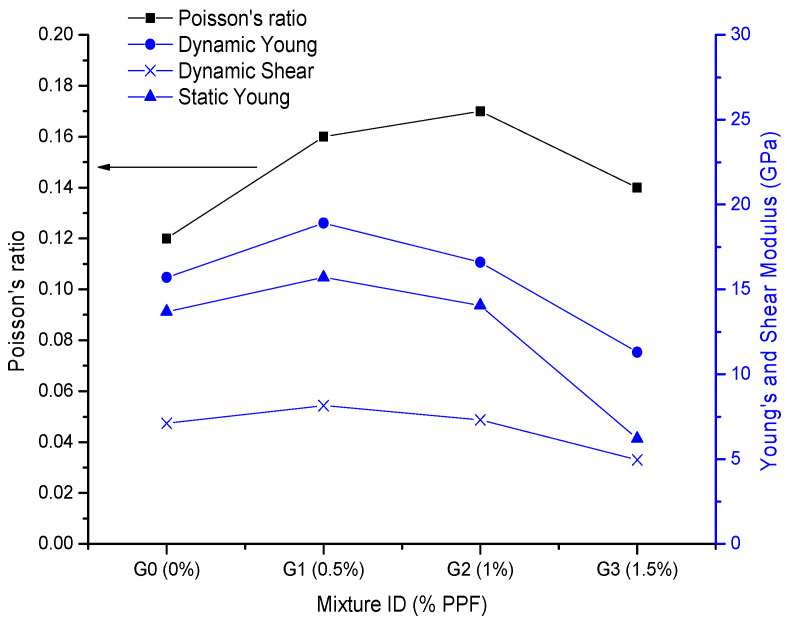
Elastic constants at 14 days.

Regarding the curing time, Table 3 shows that the elastic properties were significantly improved at 7 days compared with 1 day; however, from 7 to 14 days they did not further significantly increase. Hence, the study was limited to 14 days of curing. At 14 days, the Young’s and shear moduli were the highest for the 0.5% PPF samples, while the highest Poisson’s ratio was achieved with 1% PPF (Figure 4). Notably, there was a close relationship between Es and Ed for the G0, G1 and G2 samples.

The improved elastic behavior of G1 and G2 was correlated with good fiber dispersion and an acceptable fiber–matrix interaction. However, the drastic reduction in the elastic properties of G3 was due to the induced porosity in the composite because of fiber agglomeration due to poor dispersion [23].

Puertas et al. [26] found that the Es of a FA-based geopolymer was reduced by 17% by adding 1% PPF. Two reviews [7,23] found insufficient data on the Es values of geopolymeric composites containing PPF; nevertheless, the reviews stated that when fiber is included, the elastic modulus is mainly affected by the fiber stiffness and porosity of the composite. It was also found that although several equations have been proposed for estimating the elastic modulus using compressive or flexural strengths, additional investigations are required to confirm their applicability to different geopolymer composites.

Regarding Poisson’s ratio, Table 3 shows that the values increased as the fiber content increased (up to 1%), and they decreased as the curing time increased. At 14 days, Poisson’s ratio for the control sample was 0.12 and adding fiber increased it by 0.14–0.17 (Figure 4). Poisson’s ratios of between 0.08 and 0.22 have been found for plain and steel-fiber-reinforced geopolymers [23]. However, the experimental data for Poisson’s ratio have rarely been reported; they are typically estimated using equations available for cementitious composites. Therefore, more research is required to estimate Poisson’s ratios for PP-fiber-reinforced geopolymers.

**Figure 5:**
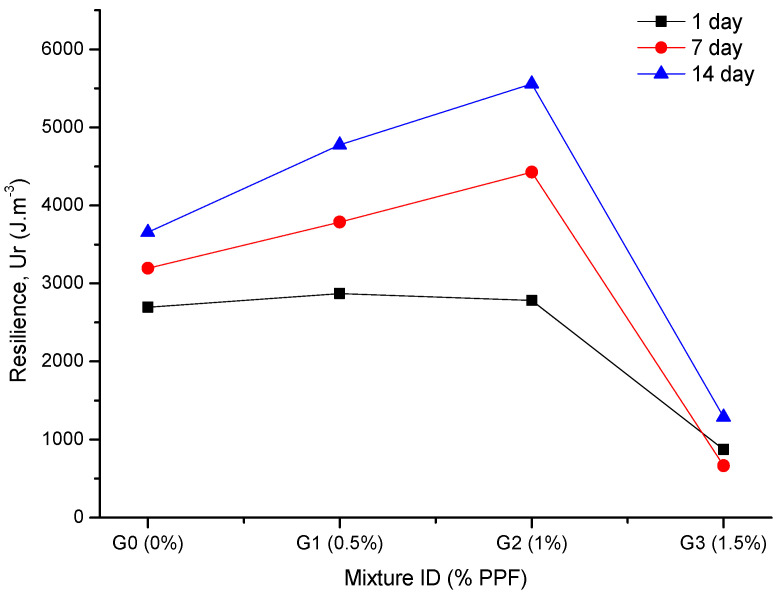
Effect of PPF content on resilience.

Most structures suffer only limited loads during their service lives. The materials are thus mostly in an elastic state, and resilience (Ur) is a key parameter to understanding the ability of composites to absorb energy when deformed elastically and to release that energy upon unloading. To observe the effect of fiber addition on the energy, which a geopolymeric composite can absorb and release under an external axial load, the Ur was determined by integrating the stress–strain curves from zero to yield stress (33% of compressive strength). The Ur values are shown in Table 3 and Figure 5. They show that the addition of 0.5% PPF (G1) and 1.0% PPF (G2) improved the Ur compared with the control sample (G0). For example, at 14 days, the Ur of G1 and G2 increased by 43% and 30%, respectively, with respect to G0. For G3, the Ur decreased by 51% compared with G0. Additionally, for samples containing up to 1% PPF, there was a significant increase in resilience with increasing curing time (Figure 5).

The increased energy absorption observed for the G1 and G2 composites is related to the fiber–matrix interaction mechanism. This is directly related to the amount of energy that is absorbed in encounters between cracks and fibers; that is, when a crack reaches a fiber, some energy is required for debonding and for the crack to pass through the fiber. This energy is required because of the integral adhesion and mechanical interactions between fiber and matrix. Previous research [17,21] has shown that energy absorption increases as the PPF content increases.

**Figure 6:**
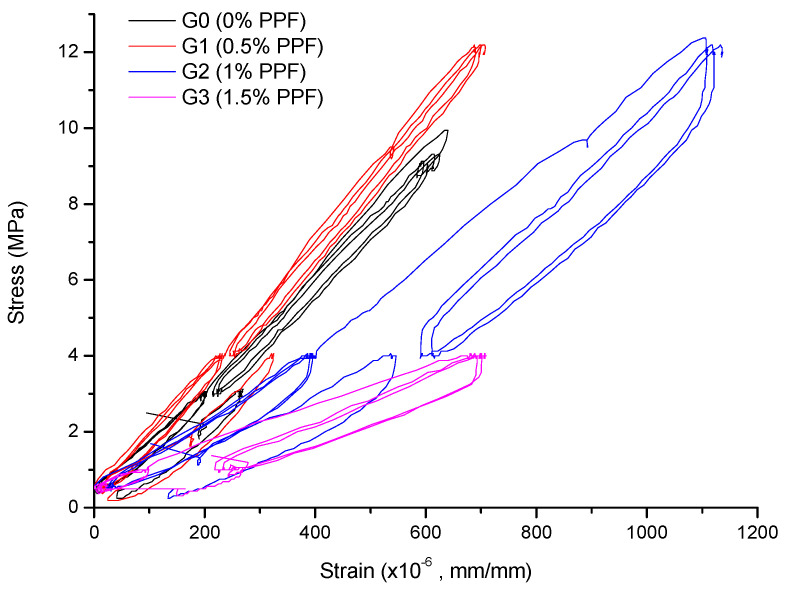
Stress–strain curves in the elastic range at 14 days.

Figure 6 shows the stress–strain curves in the elastic range at 14 days. Only the G2 composite showed a significantly altered failure behavior from a brittle mode to a ductile mode. This might be because of the strong fiber–matrix interaction, which is directly related to the amount of energy that is absorbed in crack–fiber encounters. Furthermore, the yield stress of G1 and G2 was similar (approximately 12 MPa), while for G0 and G3 it was 10 MPa and 4 MPa, respectively. However, the ε_y_ for G2 (ε_y_ = 1121) was 43%, 57% and 58% greater than for G1 (ε_y_ = 786), G0 (ε_y_ = 711) and G3 (ε_y_ = 708), respectively.

### 3.3. SEM Observations

**Figure 7:**
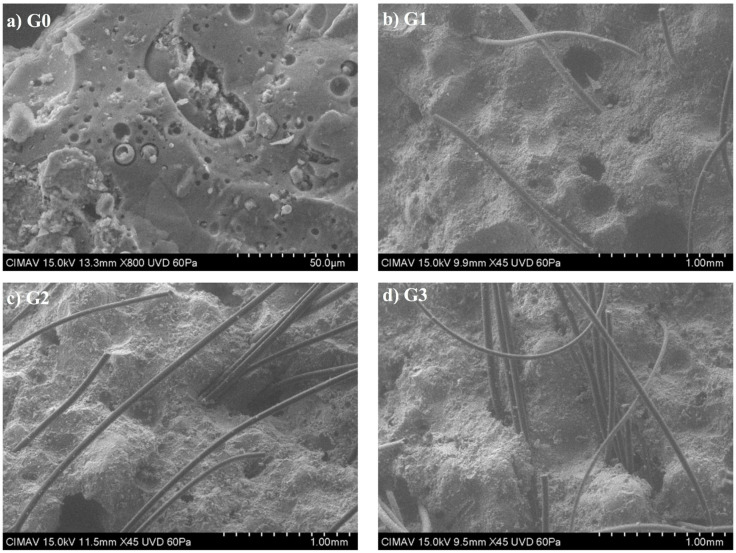
SEM micrographs of the geopolymer composites: (**a**) 0% PPF, (**b**) 0.5% PPF, (**c**) 1% PPF and (**d**) 1.5% PPF.

Figure 7a shows the basic geopolymer structure, which includes the formed amorphous geopolymeric gel, residual unreacted FA particles and varied pores (trapped air pores, capillary pores, gel pores). Figure 7b–d shows differently magnified micrographs of the PPF-reinforced geopolymer composites.

The SEM results showed that as fiber content increases, the fibers tended to agglomerate. For example, a clear agglomeration of fibers can be seen for G3 (Figure 7d), which influenced its drastically reduced mechanical and elastic properties. With high fiber contents, the fiber–fiber interfacial zones that are generated have weaker adhesion than the interface fiber–matrix adhesion. Therefore, the global adhesion capacity of the fiber system is diminished. Consequently, the mechanical properties decrease.

The drastic reduction in strength and elastic properties of the G3 (1.5% PPF) geopolymer composite can be attributed to the observed fiber agglomeration observed in Figure 7d because of poor fiber dispersion, which was noticed in the difficulties of casting and compaction of the fresh material. Fiber agglomeration due to poor dispersion causes weak fiber–matrix interactions.

**Figure 8:**
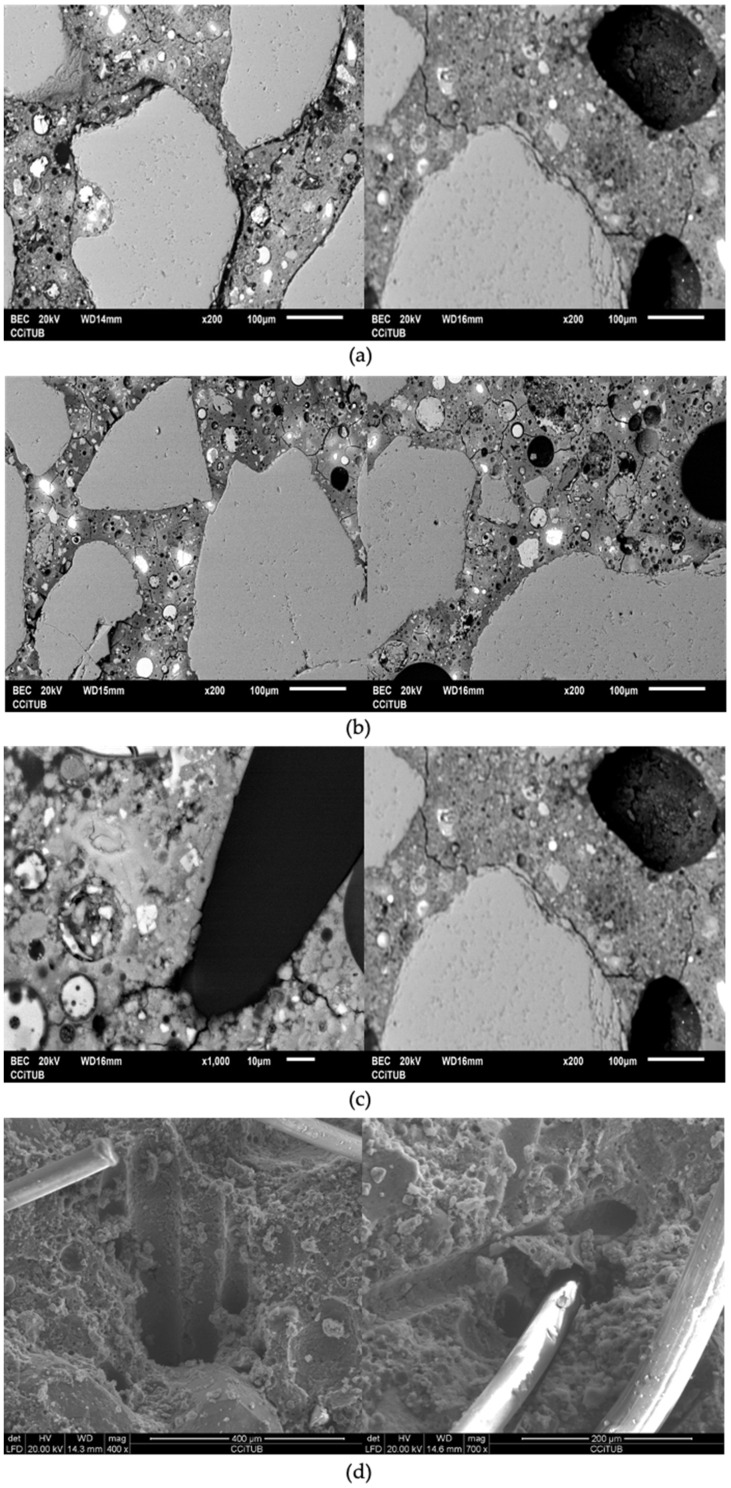
Interaction mechanism between the fibers and geopolymeric matrix; (**a**) Cracking initiation; (**b**) Crack propagation; (**c**) Crack propagation delay; (**d**) Debonding or fiber breakage.

The interaction mechanism between the fibers and geopolymeric matrix can be seen in Figure 8. This mechanism (commonly called a “bridging mechanism”) is directly related to the amount of energy that is absorbed in encounters between cracks and fibers; that is, when a crack reaches a fiber, energy is needed for debonding and passing for the crack to pass through the fiber [34]. The increased energy absorption explains the higher elastic performance of the geopolymeric that has good fiber dispersion compared with the geopolymeric composite without fibers.

The interaction mechanism between the fibers and geopolymeric matrix is described as follows. First stage cracking begins in the sand–matrix interface (more porous area due to the wall effect) and areas of high porosity in the matrix (Figure 8a). Second stage cracks are propagated through the weakest areas of the matrix (unreacted FA-rich areas and porous areas) (Figure 8b). Third stage occurs when the crack reaches the front of the fiber (Figure 8c), its propagation is delayed until the fiber–matrix adhesion is overcome (fiber–matrix debonding) or until the fiber breaks (Figure 8d). Previous research has theoretically explained this mechanism [18].

The main finding of this research is that it was possible to significantly improve the resistance to compression and bending and the elastic behavior of a fly ash-based geopolymer by incorporating 0.5% and 1.0% PPF. This study complements recent findings because although several equations were proposed for estimating the elastic modulus using compressive or flexural strengths, further investigations were required to confirm their applicability for different geopolymer composites. In addition, a recent review [23] found that experimental data relating to Poisson’s ratio and resilience have rarely been reported. They are typically only estimated using equations available for cementitious composites. Therefore, more research is required to estimate the elastic modulus, Poisson’s ratio and resilience of fiber-reinforced geopolymers.

## 4. Conclusions

This research was carried out to clarify three fundamental aspects of geopolymeric composites enhanced by the use of polypropylene fibers: their mechanical strength, elastic deformation behavior and resilience. To understand the underlying trends in these parameters, the fiber–matrix interaction mechanism was investigated by means of SEM. The most significant conclusions were as follows.

The compressive and flexural strengths significantly improved when the curing time was prolonged to 1 and 7 days; however, a curing time longer than 7 days reduced these strength parameters. The changes in the strength parameters presented similar curves for the control sample and the samples containing polypropylene fibers (in the range of 0.5 to 1.0% PPF) in relation to the speed of strength acquisition (i.e., maturity). For the optimal samples (0.5% and 1.0% PPF, at 14 days), the strength increased by 23% and 25%, respectively, compared with the control. Regarding the 1.5% PPF sample, the capacity to acquire strength was significantly reduced for all curing times (60% compared with control). This behavior is probably linked to various factors studied in previous investigations, such as the components of the composite, the mixing method, the type and content of the fibers, the curing conditions and even the size and type of sample.

Regarding the flexural strength, a significant growth rate was determined for the samples containing fibers. At 7 days, the 0.5% and 1.0% PPF samples showed a 27% and 65% increase in flexural strength compared with the control sample, while, at 14 days, these were 31% and 61%, respectively. As for the compressive strength, the use of 1.5% PPF resulted in a significant loss at all curing times, ranging from −24% to −46% compared with control. The increase in flexural strength is explained by the presence of PPF, which makes the geopolymer matrix resistant to cracking when it deforms elastically under the mechanical effect of stress. By contrast, the loss of flexural strength seen for the 1.5% PPF sample can be attributed to imperfect fiber dispersion, which was corroborated by the difficulties experienced in pouring and compacting the fresh material, leading to poor fiber distribution. Fibers that are poorly distributed throughout the matrix create excessive porosity during mixing.

The use of 0.5% and 1.0% PPF improved the deformation behavior. For 0.5% PPF at 7 days, the Es and ε_y_ were improved by 25% and 65%, while for 1.0% PPF, the Es and ε_y_ were improved by 14% and 72% compared with the control sample, respectively. The 1.5% PPF sample showed drastic reductions in these properties. The behavior of the 0.5% and 1.0% PPF samples correlates with good fiber dispersion and acceptable fiber–matrix interaction. By contrast, the poorer results of the 1.5% PPF sample were related to the induced porosity in the composite material due to fiber agglomeration and poor dispersion.

The addition of 0.5% and 1.0% PPF improved the resilience of the composites. For the 14 days curing time, the 0.5% and 1.0% PPF samples showed 43% and 30% increased resilience, respectively, compared with the control, which was due to the fiber–matrix interaction mechanism. For the 1.5% PPF sample, the resilience was reduced by 51% compared with the control.

Finally, the SEM observations allowed us to establish that the use of PPF in geopolymers improves their behavior by strengthening the fiber–matrix interface and increasing the mechanical and deformation capacities. However, there is a “saturation point” of the PPF content above which its agglomeration occurs. In such a case, the fiber–fiber adhesion is weaker than the matrix–fiber adhesion. Additionally, undesirable porosity is formed during the kneading phase. These two adverse effects reduce the performance of the geopolymer.

## Figures and Tables

**Table 1:** XRF analysis of the fly ash.

Composition	SiO_2_	Al_2_O_3_	Fe_2_O_3_	CaO	MgO	K_2_O	Na_2_O	SO_3_	TiO_2_
**FA (%)**	59.11	20.31	4.638	9.918	1.74	1.17	0.512	0.336	1.65

**Table 2:** Physical and mechanical properties of the fibers.

Material	Length (mm)	Diameter (mm)	Aspect Ratio	Shape	Tensile Strength (MPa)	Young’s Modulus (GPa)	Specific Gravity
**Polypropylene**	18	0.05	360	Mono filament	582	3.8	0.9

**Table 3:** Elastic properties at 1, 7 and 14 days.

Sample	1 Day		7 Day		14 Day		1 Day		7 Day		14 Day	
	Dynamic Young’s Modulus (Ed)						Dynamic Shear Modulus (Gd)					
	Ed (GPa)	IR (%)	Ed (GPa)	IR (%)	Ed (GPa)	IR (%)	Gd (GPa)	IR (%)	Gd (GPa)	IR (%)	Gd (GPa)	IR (%)
G0	10.9	-	15.5	-	15.7	-	4.7	-	6.8	-	7.0	-
G1	15.8	45	18.3	18	18.9	20	6.5	38	7.9	16	8.1	16
G2	16.6	52	17.6	14	16.6	6	6.7	43	7.6	12	7.1	1
G3	6.1	−44	11.1	−29	11.3	−28	2.6	−45	4.8	−29	4.9	−29
	Poisson’s ratio (μ)						Static Young’s modulus (Es)					
	μ	IR (%)	μ	IR (%)	μ	IR (%)	Es (GPa)	IR (%)	Es (GPa)	IR (%)	Es (GPa)	IR (%)
G0	0.16	-	0.14	-	0.12	-	10.42	-	13.98	-	13.68	-
G1	0.22	37	0.16	14	0.16	33	14.82	42	17.46	25	15.71	15
G2	0.24	50	0.16	14	0.17	42	16.10	55	15.88	14	14.06	3
G3	0.17	6	0.15	1	0.14	17	5.20	−50	8.20	−41	6.2	−55
	Resilience (Ur)						Elongation at yield stress (ε_y_)					
	Ur (J/m^3^)	IR (%)	Ur (J/m^3^)	IR (%)	Ur (J/m^3^)	IR (%)	ε_y_ (με)	IR (%)	ε_y_ (με)	IR (%)	ε_y_ (με)	IR (%)
G0	2695	-	3194	-	3654	-	410	-	440	-	711	-
G1	2871	7	3787	19	4775	31	712	74	725	65	786	11
G2	2784	3	4426	39	5557	52	300	−27	756	72	1121	58
G3	872	−68	664	−79	1290	−65	670	63	285	−35	708	−0.4

Note: IR (Improvement Ratio, %) = [(value of sample − value of control sample G0)/value of control sample G0] × 100.
